# Work unit level personnel working hours and the patients’ length of in-hospital stay–An administrative data approach

**DOI:** 10.1371/journal.pdig.0000265

**Published:** 2023-05-30

**Authors:** Oxana Krutova, Jenni Ervasti, Marianna Virtanen, Laura Peutere, Mikko Härmä, Annina Ropponen

**Affiliations:** 1 Finnish Institute of Occupational Health, Helsinki, Finland; 2 School of Educational Sciences and Psychology, University of Eastern Finland, Joensuu, Finland; 3 Division of Insurance Medicine, Department of Clinical Neuroscience, Karolinska Institutet, Stockholm, Sweden; Yonsei University College of Medicine, REPUBLIC OF KOREA

## Abstract

Administrative data accumulating daily from hospitals would provide new possibilities to assess work shifts and patient care. We aimed to investigate associations of work unit level average work shift length and length of patient in-hospital stay, and to examine the role of nurse-patient-ratio, year, night work, age, work units and working hours at the work units for these estimations. The data for this study were based on combined administrative day-to-day patient and pay-roll based objective working hour data of employees of one hospital district in Finland for 2013–2019. Three patient measures were calculated: the overall length of in-hospital stay, the length of in-hospital stay before a medical procedure and the length of in-hospital stay after a medical procedure. A Generalized Linear Mixed Model (GLMM) with multivariate normal random effects was used with Penalized Quasi-Likelihood for relative risk ratios (RR) with 95% confidence intervals (CI). The results showed that compared to <8 hours work shifts, 8–10 hours work shifts were associated with an increased likelihood of overall length of in-hospital stay (RR 1.16, 95%CI 1.15, 1.16), and the length of in-hospital stay after a medical procedure (RR 1.28, 95%CI 1.27, 1.30). The >10 hours work shifts were associated with a decreased likelihood of the overall length of in-hospital stay (RR 0.94, 95% CI 0.94, 0.95) and length of in-hospital stay after a medical procedure among all occupations (RR 0.94, 95% CI 0.92, 0.97). These associations retained the magnitude and direction in the models additionally adjusted for work, employee, and patient characteristics, and the associations were weaker for nurses than among all occupations. To conclude, compared with the standard work shifts, 8–10 hours work shifts seem to be associated with longer, and >10 hours work shifts with shorter length of in-hospital stay. Administrative data provides feasible possibilities to investigate working hours and length of in-hospital stay.

## Author summary

To growing extent, health care sector collects routinely administrative data for day-to-day patient and employee related activities, e.g., hospital admissions, length of in-hospital stay, or employee working hours. These data provide possibilities not only for research, but also for decision-making to increase understanding and improve personnel management, workflow, and usability of health care systems. Our access to combined administrative day-to-day patient and pay-roll based objective working hour data of employees of one hospital district in Finland for 2013–2019 enabled us to investigate the associations between employee’s work shift length at work unit level and patients’ length of in-hospital stay. We observed that 8–10 hours work shift length was associated with longer length of in-hospital stay of patients, whereas work shift length exceeding 10 hours was linked with shorter in-hospital stay. We found weaker associations for registered nurses and practical nurses compared to all occupations. These findings indicate that administrative data provides possibilities to investigate working hours and in-hospital stay length and potentially such data can be utilized even in hospital level.

## Introduction

Nurse staffing is a crucial factor for the length of in-hospital stay [1,2]. On the other hand, length of stay is an important indicator of the efficiency of hospital management [3,4]. Increasing number of on-duty health care personnel may shorten the length of stay [5], and higher average working hours per patient day have predicted shorter length of in-hospital stay [6]. A greater number of working hours per patient day has been shown to be linked with shorter length of stay also in Finland [7,8]. A higher number of night-time staffing and a higher skill mix have also been shown to be associated with shorter length of stay [9]. Until now, some data-mining studies using patient-related characteristics from administrative data have predicted the length of stay and shown that discharge delay time, operation frequency, presence of comorbidities and insurance type modify the length of average hospital stay [3,10]. However, studies are few with large-scale data of combined employee and patient data across several years [2,11,12].

Extended working hours and long work shifts may indicate high workload. Two literature reviews have shown that extended (>8 hour) working hours are associated with patient safety outcomes, such as medical errors [13,14]. These findings are in line with a cross-sectional survey study of nurses in acute hospitals [15]. Aligned with these, a previous study has linked extended working hours of registered nurses, licensed practical nurses, and certified nursing assistants with higher rates of unplanned rehospitalization and emergency department visits (both for short <101 days, and long >100 days length of stays) [16]. However, also opposite findings exists as an increase in working hours among nurses was associated with shorter length of stay in a one-year study carried out in an acute care hospital [7]. Among the measures of workload, a one-hour decrease in registered nurse staffing level per day has been associated with poorer quality of care based on compilation of 15 nursing home quality of care indicators [17]. Furthermore, the nurse-to-patient ratio (NPR) has been shown to be associated with the care process. An increase in the NPR may reduce the length of stay while a decrease in NPR may increase the frequency of patients leaving before treatment completion [18]. However, there is a gap in previous research focusing on nursing occupations, as shift length has not been studied in relation to the length of stay while accounting both nurses and other staff [19].

Today, administrative data that is routinely collected in health care sector for day-to-day activities of health care, e.g., hospital admissions, length of in-hospital stay, or employee working hours provide rich, powerful datasets that can be used not only for scientific studies but also for decision-making to improve health care systems [20–22]. As in previous studies [8,18] using administrative data, in this study we had an opportunity to use data pools that include real-time records of patient characteristics in each hospital ward (e.g., daily information on the number of treated patients and length of in-hospital stays) linked with digital payroll-based records of health care personnel’s daily working hours, including all work shifts, absences from work and sociodemographic characteristics of all employees in all work units [23], which enabled an objective and timely assessment of working hours and length of in-hospital stay.

## Objective

This study aimed to investigate whether administrative data can be used to estimate the associations between work unit level average work shift length and (a) length of patient in-hospital stay; (b) length of in-hospital stay before a medical procedure; and (c) length of in-hospital stay after a medical procedure. Moreover, we investigated the role of various influential factors for these estimations including NPR, year, night work, employee and patient age, work units and working hours at the work units.

## Data and methods

The data for this study were based on combined administrative patient data (Auria) and pay-roll based objective working hour data of employees (Titania) of one hospital district in Finland for the years 2008 to 2020. These two databases were aggregated at day and work unit-level through the first day (date) of in-hospital stay both for patient data and for working hour data of employees. For each employee, the working hour data included work shift length and the shift starting and ending times. Based on job titles according to the International Standard Classification of Occupations (ISCO) 2010, the employee data of this study included 48% registered nurses, 10% practical nurses (these were later combined into one group and later called “nurses”), 8% administrative personnel, 7% nursing assistants, and rest were other occupations. For statistical analyses, we analyzed nurses and all occupations separately. For each patient, the data included the date of admission, the date of discharge and where relevant, the date of a medical procedure. Both working hour data and patient data included the work unit code for matching. The patient data contained 631,326 patient visits. In the aggregated data including both patient and employee data, each row contained a patient’s day in in-hospital stay matching with the average shift length per work unit and per day. We restricted the data to include only the patients who had been for at least one day in hospital care (difference between the time of start and the time end of admission is ≥ 24 hours, i.e., short visits due to outpatient care and visits without staying overnight were excluded). Then we further excluded from the analysis the work units that contributed less than 1% of the data.

**Fig 1:**
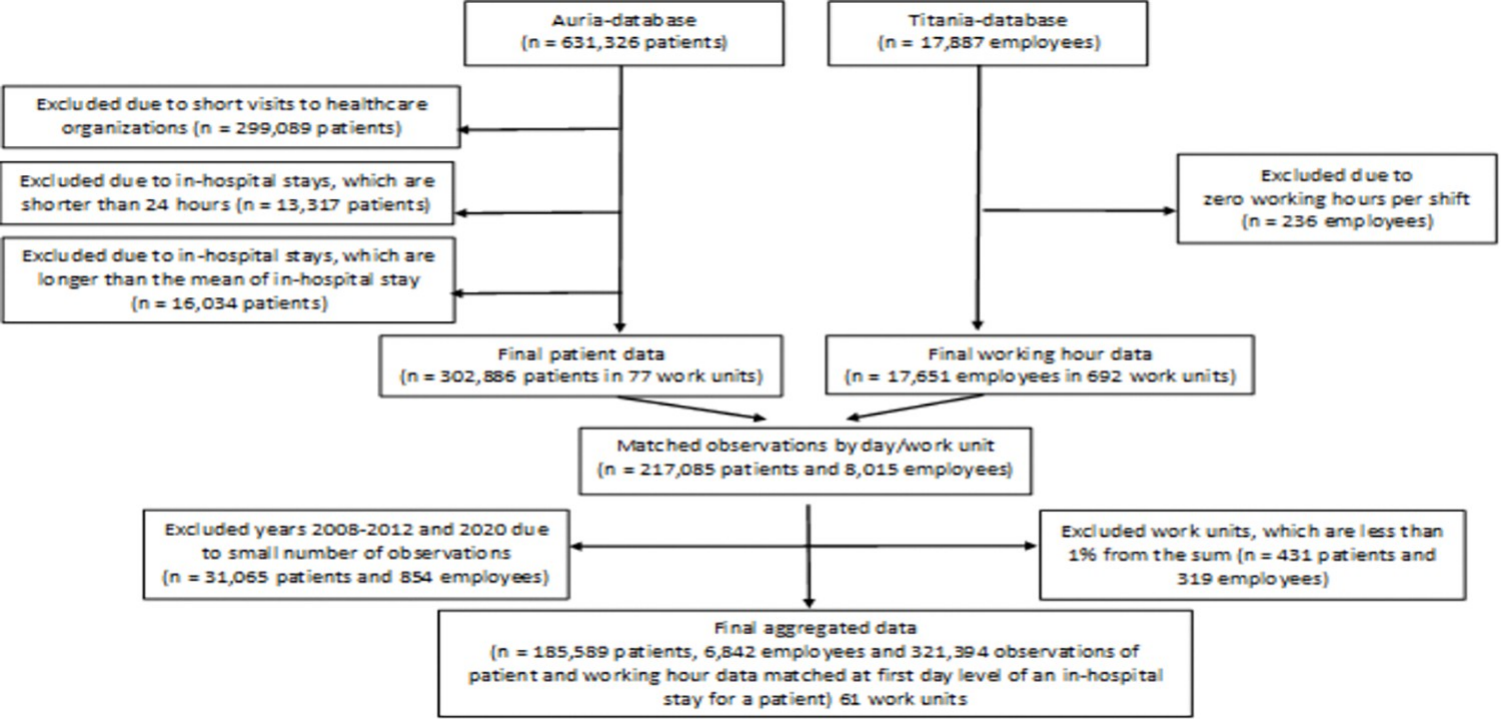
A flow chart of data selection for overall length in-hospital stay.

The flow chart for data selection and combination of patient data with employee data is shown in Fig 1. We firstly estimated the overall length of in-hospital stay. After that we estimated the wait time of the patients for a medical procedure (length of in-hospital stay before a medical procedure) and the time after a medical procedure had been done. The type of medical procedure, i.e., whether it was a scheduled procedure or a procedure due to an urgent change in patient status was not known. Since not all the patients have had a medical procedure, the samples for these two measures were smaller. This study was fully based on administrative register data that the hospital district had permitted the access. Research using such data does not need to undergo review by an ethics committee according to Finnish legislation (Medical Research Act).

### Study outcome

The length of in-hospital stay was estimated as the number of in-hospital patient days (in which patients stayed overnight), as previously [3,24]. For the length of in-hospital stay, three measures were applied. We firstly estimated the length of in-hospital stay (mean 6.40 days, standard deviation (SD) 15.49 days, possible range 1–2618 days). After that we estimated the wait time of the patients for a medical procedure (length of in-hospital stay before a medical procedure, mean 4.83 days, SD 12.78, possible range 1–1711 days). Finally, we estimated the time after a medical procedure had been done (mean 9.05 days, SD 19.93, possible range 1–907 days), i.e., length of in-hospital stay after a medical procedure. The mean length of in-hospital stays (e.g., 6.40 days, 4.83 days and 9.05 days) was then used for estimating the upper bound of the length of in-hospital stay measures for each patient (e.g., 1–6.40 days, 1–4.83 days and 1–9.05 days) as has been done before [25,26]. The length of in-hospital stay measures were calculated for the all in-hospital stays (possible range 1–321,394) and as a sensitivity analysis, also for the first in-hospital stay only.

### Main exposure of interest

As the main exposure of interest, we calculated the average length of work shifts (calculated as the mean for all the days during which the patient was treated in the hospital) for each length of in-hospital stay measure at work-unit level, i.e., separately for the whole hospital stay, the time before procedure and the time after procedure. We calculated the working hours separately for nurses (i.e., registered nurses and practical nurses) and for all occupations. For the statistical analyses, we categorized the average length of work shifts to <8 hours vs. 8–10 and >10 hours at work-unit level. The <8 hours shift length was selected as reference category based on the limits of the earlier research indicating increased risk for employee health and wellbeing [27,28], and patient outcomes [7,8]. In general, the working hours in hospitals are irregular and period based [28], guided by the municipal health and social sector’s collective agreement [29] that sets working hours per each three-week period. Currently the working hours should be at 114 hours 45 minutes/3-week period and the same rule has applied earlier [30]. Shift length should be 10 hours at maximum, but there is no limit to the weekly working hours if the obligatory weekly rest period (minimum 24 hours and averagely 35 hours in two calendar weeks) is followed.

### Covariates

We controlled the models for the average nurse-to-patient ratio (NPR) at work-unit level for each of the three length of in-hospital stay measures. NPR was calculated only for nurses. The other covariates were the proportion of night shifts of all shifts during one in-hospital stay at work-unit level, the sum of the operational working hours per day at work-unit level during one in-hospital stay for each patient, average age of employees at the first day of in-hospital stay, year of in-hospital stay, age of patients and to account the changes at the ward-level, work units at which patients have been firstly assigned at the first day of in-hospital stay.

### Statistical analyses

First, descriptive statistics including means with standard deviations (SD) and proportions were estimated. Then we, used a Generalized Linear Mixed Model (GLMM) with multivariate normal random effects, using Penalized Quasi-Likelihood, to estimate random effects in addition to the usual fixed effects. We used 2-level model, which contains level 1 (level of in-hospital stay) and level 2 (a person, who could have more than 1 in-hospital stay). The Poisson distribution was used as the three outcome variables (overall length of in-hospital stay, length of in-hospital stay before a medical procedure and length of in-hospital stay after a medical procedure) are count variables. The second component of GLMM, a linear predictor, was the average working hours per work shifts during one in-hospital stay. The R (version 4.0.5) was used for all analysis. We utilized MASS and nlme libraries for the GLMM analyses. We transformed the log rate ratios of GLMM to rate ratios (RR) with 95% confidence intervals (CI) via exp(log rate ratios) and exp(log rate ratios ± 1.96 × SE) functions. R (version 4.0.5) was used for all analyses.

## Results

Table 1 displays descriptive statistics for the final data derived from 61 work units and patients’ first in-hospital stays only (n = 6,780 employees). The mean length of in-hospital stay per a patient (n = 185,589 patients) was 2.5 days (range 1–6.4), 1.5 days for length of in-hospital stay before a medical procedure (range 1–4.8), and 2.7 days for length of in-hospital stay after a medical procedure (range 1–9.1) (Table 1). On average, employees had 8.3 working hours per shift at work unit level (8.5 hours for length of in-hospital stay before a medical procedure and 8.4 hours for length of in-hospital stay after a medical procedure). NPR was 0.6 for overall length of in-hospital stay and length of in-hospital stay after a medical procedure, but 0.9 for length of in-hospital stay before a medical procedure. The average proportion of night shifts (of all shifts) was 13% for overall length of in-hospital stay and length of in-hospital stay after a medical procedure and 14% for length of in-hospital stay before a medical procedure.

**Table 1:** The descriptive statistics for length of shift (working hours per shift), outcomes of interest and covariates in the sample including all hospital stays for a patient (means with standard deviation (SD)).

	All in-hospital stays					
	Overall length in-hospital stay (n = 321,394, 1–61 stays)		Length of in-hospital stay before a medical procedure (n = 60,112, 1–23 stays)		Length of in-hospital stay after a medical procedure (n = 98,903, 1–23 stays)	
	n = 185,589 patients		n = 46,938 patients		n = 71,959 patients	
	n = 6,780 employees		n = 5,379 employees		n = 5,451 employees	
	**Mean**	**SD**	**Mean**	**SD**	**Mean**	**SD**
Length of in-hospital stay (days) of patients	2.5	1.5	1.5	0.8	2.7	1.9
**Exposure**						
Working hours per work shift at work unit level for employees (hours)	8.3	0.8	8.5	0.9	8.4	0.7
	**n**	**%**	**n**	**%**	**n**	**%**
**Length of work shift at work unit level for employees**						
<8 hours work shifts	2,101	31	1,216	23	1,310	24
8–10 hours work shifts	4,538	66	4,015	74	3,985	73
>10 hours work shifts	203	3	148	3	156	3
	**Mean**	**SD**	**Mean**	**SD**	**Mean**	**SD**
**Covariates**						
Employee’s age (years)	42.1	4.1	41.7	2.6	41.9	3.0
NPR at work unit level for employees (ratio)	0.6	0.8	0.9	1.5	0.6	0.8
Proportion of night shifts (of all shifts) at work unit level for employees (%)	12.7	16.5	14.0	16.0	13.3	15.4
The sum of the operational working hours per day at work-unit level for employees (hours)	409.6	245.0	496.9	256.1	472.2	241.2
Patient’s age (years)	49.9	27.6	55.8	22.7	52.9	22.5
	**n**	**%**	**n**	**%**	**n**	**%**
**Year**						
2013 (n of employees)	1,085	16	597	11	633	12
2014 (n of employees)	890	13	433	8	512	9
2015 (n of employees)	873	13	922	17	929	17
2016 (n of employees)	800	12	838	16	786	14
2017 (n of employees)	808	12	767	14	755	14
2018 (n of employees)	1,194	17	789	15	813	15
2019 (n of employees)	1,192	17	1,033	19	1,023	19

In comparison to work shifts <8 hours in work units, work shift length of 8–10 hours was associated with a longer duration in all three indicators of in-hospital stay among all occupations as well as among nurses only (Table 2). However, this association was stronger for the length of in-hospital stay after a medical procedure and for all occupations in comparison to nurses. The work shift >10 hours was associated with a shorter in-hospital stay (RR 0.85, CI 0.83–0.86) and a shorter in-hospital stay after a medical procedure (RR 0.88, CI 0.85–0.91) (Model 3, all occupations). For nurses, however, this association was slightly smaller. In general, these associations retained the magnitude and direction in the additionally adjusted models 4 and 5 (NPR or with operationalized working hours in the work unit). As a sensitivity analysis, the average shift length was tested as continuous measure. The results for each one-unit increase of working hours indicated lower odds for overall hospital stay, whereas the results were non-significant for length of stay before or after a medical procedure (S1 Table).

**Table 2:** Generalized Linear Mixed Models (GLMM) for rate ratios (RR) with 95% confidence intervals (CI) for the associations between the average working hours per shift during one in-hospital stay and three length of in-hospital stay measures for all in-hospital stays in 2013–2019.

Working hours per shift in a work unit	Overall length of in-hospital stay									
	Model 1^a^		Model 2^a^		Model 3^a^		Model 4^a^		Model 5^a^	
	RR	95%CI	RR	95%CI	RR	95%CI	RR	95%CI	RR	95%CI
All occupations										
<8 hours	1.00	ref	1.00	ref	1.00	ref	1.00	ref	1.00	ref
8–10 hours	**1.16**	**1.15, 1.16**	**1.11**	**1.11, 1.12**	**1.11**	**1.10, 1.11**	**1.11**	**1.10, 1.11**	**1.11**	**1.11, 1.12**
>10 hours	**0.86**	**0.85, 0.87**	**0.86**	**0.85, 0.87**	**0.85**	**0.83, 0.86**	**0.83**	**0.81, 0.84**	**0.86**	**0.85, 0.87**
Nurses										
<8 hours	1.00	ref	1.00	ref	1.00	ref	1.00	ref	1.00	ref
8–10 hours	**1.05**	**1.05, 1.06**	**1.04**	**1.04, 1.05**	**1.03**	**1.02, 1.03**	**1.03**	**1.02, 1.03**	**1.04**	**1.03, 1.04**
>10 hours	**0.94**	**0.94, 0.95**	**0.97**	**0.96, 0.98**	**0.91**	**0.90, 0.92**	**0.90**	**0.89, 0.91**	**0.94**	**0.93, 0.95**
**Length of in-hospital stay before a medical procedure**										
All occupations										
<8 hours	1.00	ref	1.00	ref	1.00	ref	1.00	ref	1.00	ref
8–10 hours	**1.13**	**1.12, 1.14**	**1.10**	**1.09, 1.11**	**1.09**	**1.08, 1.10**	**1.09**	**1.08, 1.10**	**1.10**	**1.08, 1.11**
>10 hours	**1.10**	**1.08, 1.12**	1.00	0.98, 1.02	0.99	0.97, 1.01	**0.97**	**0.95, 0.99**	1.00	0.98, 1.02
Nurses										
<8 hours	1.00	ref	1.00	ref	1.00	ref	1.00	ref	1.00	ref
8–10 hours	**1.10**	**1.09, 1.11**	**1.07**	**1.06, 1.09**	**1.06**	**1.05, 1.08**	**1.06**	**1.05, 1.08**	**1.07**	**1.05, 1.08**
>10 hours	**1.10**	**1.08, 1.12**	**1.03**	**1.01, 1.05**	1.00	0.98, 1.02	0.97	0.95, 1.00	**1.02**	**1.00, 1.05**
**Length of in-hospital stay after a medical procedure**										
All occupations										
<8 hours	1.00	ref	1.00	ref	1.00	ref	1.00	ref	1.00	ref
8–10 hours	**1.28**	**1.27, 1.30**	**1.21**	**1.20, 1.22**	**1.21**	**1.20, 1.22**	**1.21**	**1.20, 1.23**	**1.22**	**1.21, 1.24**
>10 hours	**0.94**	**0.92, 0.97**	**0.88**	**0.86, 0.91**	**0.88**	**0.85, 0.91**	**0.85**	**0.82, 0.88**	**0.89**	**0.86, 0.91**
Nurses										
<8 hours	1.00	ref	1.00	ref	1.00	ref	1.00	ref	1.00	ref
8–10 hours	**1.12**	**1.11, 1.13**	**1.11**	**1.10, 1.12**	**1.10**	**1.09, 1.11**	**1.10**	**1.09, 1.12**	**1.11**	**1.10, 1.12**
>10 hours	1.02	1.00, 1.03	1.01	0.99, 1.03	**0.94**	**0.92, 0.96**	**0.95**	**0.93, 0.97**	**0.98**	**0.95, 1.00**

^a^ Model 1 unadjusted, Model 2 adjusted for covariates employee’s age, patient’s age, years and work unit, Model 3 adjusted for covariates employee’s age, patient’s age, night work, years and work unit, Model 4 was as Model 3 and additionally adjusted for nurse-to-patient ratio, and Model 5 was as Model 3 and additionally adjusted for the sum of the operational working hours per day at work-unit level. Statistically significant RR with 95%CI in boldface.

## Discussion

This study of one hospital district in Finland with comprehensive administrative data for working hours of employees (approximate number of employees between 5,300–6,700 depending on the model) and patients’ (n = 46,000–185,000) in-hospital stays aimed to investigate whether administrative data could be used for investigations of associations between work unit level average work shift length and length of patient in-hospital stay. We found that a total of 66% of all the work shifts were between 8–10 hours, whereas for the length of in-hospital stay before and after a medical procedure 73–74% of work shifts were between 8–10 hours. Our finding of work shifts of 8–10 hours being the most common is in line with earlier studies of health care [31] but adds to the previous findings partially based on the same dataset as here [23,32–34]. On the other hand, only 3% of all the work shifts were >10 hours.

We observed an inverse J-shaped association between work shifts and in-hospital stay length. The comparison with a standard shift length (<8 hours); an average shift length of 8–10 hours was associated with a longer in-hospital stay. The work shifts >10 hours were found to be associated with a shorter duration of in-hospital stay and shorter in-hospital stay after a medical procedure. The >10 hours work shifts were associated with shorter overall stay even when adjusted for the average operational working hours. These findings partly align with the previous studies, indicating a negative effect of longer work shifts on safe care and missed nursing care, lower productivity and efficiency [35]. Earlier studies have also shown that work shifts (>8 hours) are associated with patient safety [13–15]. Besides the length of stay, earlier studies have indicated that longer working hours may be linked with higher rates of unplanned rehospitalization and emergency department visits [16]. Instead, the use of ≥12 hours work shifts has been associated with shorter working weeks [28] in period-based work where the average working hours are balanced every three weeks. It is thus possible that the use of >10 hours work shifts can be associated with even improved recovery of the nurses than the use of standard work shifts with a higher frequency of working days in a week.

Secondly, the use of longer work shifts may decrease the need and errors of inhouse reporting of the wards compared to shorter and more frequent shifts. Our finding is in line with previous studies which have shown that longer average working hours per patient day predicted shorter in-hospital stay [6]. Also in Finland, earlier studies showed that longer working hours per patient day were linked with shorter length of stay [7,8]. In our study, including the night shifts or other influential factors (i.e., NPR and operationalized working hours in the work unit) to the models did not affect these associations. These influential factors were accounted due to the fact that previous studies have shown that adjusting for patient churn increases nurse staffing across all units and shifts [36]. Increasing number of on-duty health care personnel [5] or a higher number of night-time staffing [9] were also assumed to shorten the length of stay. Earlier, an increase in the NPR were shown to reduce the length of stay [18], although we did not find this effect.

Administrative data that is routinely collected in hospitals’ day-to-day activities, and hospital admissions, length of in-hospital stay and employee working hours provided comprehensive data for this study. Hence, we utilized data that included real-time records of patient’s admissions and age in each hospital ward (e.g., daily information on the number of treated patients and length of in-hospital stays) with digital payroll-based records of health care personnel’s daily working hours [8,18,23] enabling matching at work unit level. Therefore, the strengths of this study included the possibility to investigate work shift length in association with the patients’ length of stay that has been rarely studied before [7,8]. Another strength is the use of large administrative data of employees and patients for the period of seven years. To the best of our knowledge, studies are few with large-scale data of combined employee and patient data across several years [2,11,12].

The main limitation of this study is lack of specificity. In this study, the observation period was limited to seven years while the patient data were restricted to the patients who had been for at least one day including overnight stay in hospital care. In addition, we had to exclude some very small work units with few (<1%) observations. Yet another limitation might be related to the use of measures of in-hospital stay which were restricted to the mean. This dilutes the effect of the very long treatment periods form the analyses although inclusion of all lengths would have resulted inclusion of some potential outliers and larger variations potentially affecting the results. The study was restricted to Finland, and we lacked details of nurses’ education and skill level, and patients’ diagnosis and disease severity were only accounted with patient’s age and work unit, a proxy for ward type. Thus, further studies are needed to replicate our findings in other settings and while adding more strict evaluation of patient data. However, our results should be more likely under- than over-estimated, and at least indicates that these types of administrative data can be used for these purposes. Yet another limitation might be associated with is the finding of the >10 hours work shifts with a shorter duration of in-hospital stay. Although we controlled several confounding factors (i.e., factors associated both with employees and patients), we cannot exclude potential (measured or unmeasured) bias related to the measures of this study or other confounding factors than available in this study (e.g., work unit or patient characteristics).

## Conclusions

To conclude, work shifts of 8–10 hours were associated with longer length of in-hospital stay of patients. In turn, work shift length of >10 hours was linked with shorter length of in-hospital stay. The associations were weaker for nurses than for all occupations. Hence administrative data provides feasible possibilities to investigate working hours in association with patient stays in hospitals.

## Supporting information

S1 Table Generalized Linear Mixed Models (GLMM) for odds ratios (OR) with 95% confidence intervals (CI) for the associations between the average working hours per shift during one in-hospital stay and three length of in-hospital stay measures in 2013–2019 for all in-hospital stays and for the first hospital stay. (DOCX)
