# Supplementary material for: Work unit level personnel working hours and the patients’ length of in-hospital stay–An administrative data approach
Source: PLOS Digit Health. 2023 May 30;2(5):e0000265. doi: 10.1371/journal.pdig.0000265 (PMC10228787; doi:10.1371/journal.pdig.0000265)
Supplement: S1 Table — (DOCX) [file pdig.0000265.s001.docx]

Supplemental material

**S1 Table** Generalized Linear Mixed Models (GLMM) for odds ratios (OR) with 95% confidence intervals (CI) for the associations between the average working hours per shift during one in-hospital stay and three length of in-hospital stay measures in 2013-2019 for all in-hospital stays and for the first hospital stay

| **Average working hours per shift at a work unit** | **All in-hospital stays** | | | | | |
| --- | --- | --- | --- | --- | --- | --- |
|  | **Overall length of in-hospital stay** | | | | | |
|  | **Model 1*** | | **Model 2*** | | **Model 3*** | |
|  | **OR*** | **95%CI** | **OR*** | **95%CI** | **OR*** | **95%CI** |
| All occupations | 0.99 | 0.98, 0.99 | 0.98 | 0.98, 0.99 | 0.99 | 0.99, 1.00 |
| Nurses and practical nurses | 0.99 | 0.99, 0.99 | 0.99 | 0.99, 0.99 | 1.00 | 0.99, 1.00 |
|  | **Length of in-hospital stay before a medical procedure** | | | | | |
| All occupations | 1.00 | 0.99, 1.00 | 0.99 | 0.98, 1.00 | 1.00 | 0.99, 1.01 |
| Nurses and practical nurses | 1.00 | 0.99, 1.00 | 0.99 | 0.99, 1.00 | 1.00 | 1.00, 1.01 |
|  | **Length of in-hospital stay after a medical procedure** | | | | | |
| All occupations | 1.00 | 0.99, 1.01 | 0.99 | 0.99, 1.00 | 1.00 | 1.00, 1.01 |
| Nurses and practical nurses | 1.00 | 1.00, 1.01 | 1.00 | 1.00, 1.01 | 1.01 | 1.00, 1.01 |
|  | **First in-hospital stay** | | | | | |
|  | **Overall length of in-hospital stay** | | | | | |
| All occupations | 0.99 | 0.99, 0.99 | 0.99 | 0.98, 0.99 | 0.99 | 0.99, 1.00 |
| Nurses and practical nurses | 0.99 | 0.99, 1.00 | 0.99 | 0.99, 0.99 | 1.00 | 1.00, 1.00 |
|  | **Length of in-hospital stay before a medical procedure** | | | | | |
| All occupations | 1.00 | 0.99, 1.01 | 1.00 | 0.99, 1.00 | 1.00 | 1.00, 1.01 |
| Nurses and practical nurses | 1.00 | 1.00, 1.01 | 1.00 | 0.99, 1.00 | 1.00 | 1.00, 1.01 |
|  | **Length of in-hospital stay after a medical procedure** | | | | | |
| All occupations | 1.00 | 0.99, 1.01 | 1.00 | 0.99, 1.01 | 1.01 | 1.00, 1.02 |
| Nurses and practical nurses | 1.01 | 1.00, 1.02 | 1.01 | 1.00, 1.01 | 1.01 | 1.01, 1.02 |

* Model 1 adjusted for covariates employee’s age, patient’s age, night work, years and work unit, Model 2 as Model 1 and additionally adjusted for NPR, and Model 3 as Model 1 and additionally adjusted for the sum of the operational working hours per day at work-unit level
